# Prognostic value of vascular endothelial growth factor subtypes and risk models constructed based on the common pathway of ulcerative colitis and colon cancer

**DOI:** 10.1515/med-2025-1245

**Published:** 2026-02-12

**Authors:** Shuni Chen, Haibin He, Yonghua Chen, Xunzhen Xu, Pei Liu, Feng Li

**Affiliations:** Shenzhen Hospital of Beijing University of Chinese Medicine (Longgang), Shenzhen, GuangDong, China; Department of Endocrinology, The Second People’s Hospital of Guizhou Province, Guiyang, China; Resource Institute for Chinese & Ethnic Materia Medica, Guizhou University of Traditional Chinese Medicine, Guiyang, Guizhou, China

**Keywords:** ulcerative colitis (UC), colon cancer, vascular endothelial growth factor, risk model, immunotherapy

## Abstract

**Objectives:**

Patients with long-term ulcerative colitis (UC) have a significantly increased risk of colon cancer compared to normal individuals. By analyzing the pathways of ulcerative colitis and colon cancer, we identified a common risk pathway – Vascular Endothelial Growth Factor (VEGF).

**Methods:**

Based on the Gene Set Variation Analysis (GSVA) scores of the VEGF pathway in colon cancer patients, we divided them into three groups: high, medium, and low. We performed clinical characteristic and immune-related analyses on these three groups, conducted differential gene analysis, performed univariate and multivariate Cox analyses on the differential genes to screen out gene signatures. A risk model was constructed and validated in the UC cohort. Colon cancer patients were classified into high-and low-risk groups based on risk scores, clinical characteristics, immune microenvironment, immunotherapy, and drug sensitivity were analyzed for both groups.

**Results:**

The high expression group had higher clinical staging and pathologic staging, higher immune microenvironment scores, and greater immune cell content. The risk model we constructed demonstrated good prognostic predictive ability not only for colon cancer but also for UC. The high-risk group had a lower survival rate, while the low-risk group had a better response to immunotherapy.

**Conclusions:**

These findings provide new insights into the treatment of colon cancer.

## Introduction

Colon cancer is one of the most common types of cancer in the world, and its development may be influenced by genetic factors, lifestyle factors (such as diet), environmental factors, and the presence of intestinal diseases (such as inflammatory bowel disease) [1]. According to data from the World Cancer Research Fund and the American Cancer Society, colon cancer is the third most common cancer worldwide and the second leading cause of death worldwide [2]. Ulcerative colitis (UC) is a long-term inflammatory bowel disease (IBD) that mainly affects the large intestine (colon) and rectum [3]. Long term inflammation leads to continuous cell turnover, which may lead to DNA damage and ultimately cellular mutations, creating favorable conditions for the onset of colon cancer [4]. Therefore, for UC patients, close cooperation with healthcare providers, regular monitoring, and appropriate preventive measures are crucial to reduce the risk of colon cancer [5].

Vascular Endothelial Growth Factor (VEGF) is a signaling protein produced by human cells and plays a crucial role in the formation and growth of blood vessels. VEGF mainly plays its role by promoting the proliferation and migration of endothelial cells, as well as the formation of new blood vessels (called angiogenesis). It is essential for normal physiological processes such as wound healing, embryonic development, and tissue repair [6]. The VEGF family includes various members, such as VEGF-A, VEGF-B, VEGF-C, VEGF-D, and PlGF (placental growth factor), among which VEGF-A is the most widely studied and applied member [7]. These growth factors stimulate a series of signaling pathways by binding to their specific receptors, thereby promoting the proliferation, migration, and formation of new blood vessels in endothelial cells.

Vascular endothelial growth factor is a common risk factor for UC and colon cancer [8]. By revealing the common molecular and biological pathways between UC and colon cancer, researchers can gain a deeper understanding of how these two conditions develop and progress. This understanding helps identify early markers of the disease, predict disease trajectory, and understand which factors may promote the transition from chronic inflammation to cancer.

## Methods

### Data collection

Collect gene expression and clinical information of patients with UC in the Gene Expression Omnibus (GEO) database (https://www.ncbi.nlm.nih.gov/geo/), with cohorts of GSE87466 and GSE9452. There are 108 sets of data in the GSE87466 cohorts, including 21 normal individuals and 87 patients with UC. There are 26 sets of data in the GSE9452 cohorts, including 5 normal individuals and 21 patients with UC. Download gene expression and clinical feature data related to colon cancer patients from The Cancer Genome Atlas (TCGA) database (https://portal.gdc.cancer.gov/), preprocess to obtain 465 disease group data and 41 normal group data. GSE91061 is a melanoma cohort treated with immune checkpoint inhibitors. The Imvigor-210 cohort is a clinical trial focusing on the study of Atezolizumab, an antibody against PD-L1 (programmed death ligand 1), which is used to treat urothelial cancer (including bladder cancer) [9]. We use the GSE91061 cohort and Imvigor-210 cohort to evaluate the predictive value of risk models in immunotherapy.

### Screening of differentially expressed genes (DEGs) and pathway enrichment analysis

Use R package “limma” to screen differential expressed genes (DEGs) in GSE87466 and GSE9452 and draw a volcano map, merge DEGs, and perform pathway enrichment analysis [10]. Perform Gene Set Variation Analysis (GSVA) on the expression data of colon cancer, and use GSVA score for univariate cox to screen for pathways related to patient mortality.

### Screening of gene signatures and establishment of risk model

The intersection of differentially expressed genes between high and medium groups and high and low groups is taken, and the intersection genes are subjected to univariate cox and multivariate cox to screen for gene signatures, Based on the coef values in the multivariate cox analysis of gene signatures, a risk model is constructed. The specific formula is as follows:

$$\text{Risk Score}={\text{coef}}_{\text{gene}\left[1\right]}*{\mathrm{Exp}}_{\text{gene}\left[1\right]}+{\text{coef}}_{\text{gene}\left[2\right]}*{\mathrm{Exp}}_{\text{gene}\left[2\right]}+{\text{coef}}_{\text{gene}\left[3\right]}*{\mathrm{Exp}}_{\text{gene}\left[3\right]}+\ldots +{\text{coef}}_{\text{gene}\left[n\right]}*{\mathrm{Exp}}_{\text{gene}\left[n\right]}$$

Receiver Operating Characteristic (ROC) curve used to evaluate the performance of risk models [11]. If the risk score of the sample is higher than the average risk score, it is classified as high risk group; otherwise, it is classified as low risk group. The risk model was validated in the GSE87466 and GSE9452 datasets because the VEGF pathway screening analysis was initially based on these two datasets. Therefore, validating the risk model constructed based on the VEGF pathway in these datasets further confirms the high expression levels of the VEGF pathway in these datasets. Additionally, this validation helps assess the stability and reliability of the model within the same data source.

### Construction of nomogram

Nomogram can display survival rates over time, helping researchers understand the prognosis of specific diseases and evaluate the impact of risk factors [12]. Using R package “survival” and “rms” to construct nomogram for multiple clinical features, and evaluating the accuracy of nomogram using ROC curves at one year, three years, and five years.

### Immune related analysis

Use R package “CIBERSORT” to analyze immune cell content [13]. The “estimate” package can calculate the immune and mechanistic scores of specimens using RNA seq data, thereby evaluating the purity of tumors. We use the ESTIMATE package to predict the stromal score, immune score, and tumor purity of colon cancer patients, and analyze the differences between high, medium, and low groups. Immune checkpoints are a regulatory mechanism in the immune system, consisting of a group of inhibitory receptors and ligands that can regulate the activity of T cells [14]. We collected relevant immune checkpoints information from literature and analyzed their differences. The TISDIB database (http://cis.hku.hk/TISIDB/download.php) provides genetic characteristics of 28 tumor infiltrating lymphocytes, and single-sample Gene Set Enrichment Analysis (ssGSEA) obtains the expression of each type of lymphocyte in each sample. Afterwards, perform differential expression analysis. The Cancer Immunity Cycle is a process that describes how the immune system recognizes and attacks cancer cells, emphasizing the complex interactions between cancer and the immune system [15]. We collected a set of genes related to cancer immune cycle steps from the TIP website (http://biocc.hrbmu.edu.cn/TIP/) and conducted GSVA and differential expression analysis.

### Drug sensitivity analysis and prediction of immunotherapy

“OncoPredict” is an R package that predicts drug sensitivity based on gene expression levels [16]. It can generate IC50 values for each drug based on sample gene expression levels, we calculated the correlation between 198 compounds provided by the “OncoPredict” package and risk scores, and presented the top 3 compounds with the most positive and negative correlations with risk scores based on their IC50 values.

We use gene expression data analysis on the TIDE online website (http://tide.dfci.harvard.edu/) to obtain tumor immune dysfunction, immune exculsion, and microsatellite instability expression signature (MSI), which are indicators for measuring tumor immune response [11]. IPS data from TCIA website (https://tcia.at/home) can predict the response of samples treated with immune checkpoint inhibitors PD-1/PD-L1 and CTLA-4. The Imvigor-210 and GSE91061 cohorts were used to validate the predictive results of immunotherapy. The GSE91061 and Imvigor-210 cohorts have been widely used in bioinformatics analyses for predicting immune therapy responses. For instance, Zhao et al. used the IMvigor 210 cohort to predict the response of bladder cancer patients to immunotherapy [17]. Similarly, Zhang et al. used GSE91061 to predict the response of lung adenocarcinoma patients to immunotherapy [18].

### Statistical analysis

When analyzing the GSE9452, GSE87466, GSE91061 and Imvigor-210 datasets, batch effects were corrected using the ComBat function from the ‘sva’ package to avoid bias in cross-cohort comparisons. Non parametric tests between two groups of samples are usually conducted using the Wilcox test, while non parametric tests between three or more groups are conducted using the Kruskal Wallis test. Spearman correlation analysis is used to analyze data that does not meet the normal distribution and linear relationship assumptions of Pearson correlation analysis. In survival analysis, log-rank test is used to compare whether there is a significant difference in the distribution of survival time between two or more samples. All significance analyses in this study were conducted using two-tailed test. In the process of differential gene screening using limma and Cox regression, as well as in the group comparison analysis, we applied the FDR method for multiple comparison correction. Therefore, the p-values presented in these analyses are the adjusted p-values. The specific process of this study is shown in Supplement Figure 1.

### Ethical approval

The data used in this article are from online websites, so ethical approval is not required.

## Results

### Screening of DEGs in UC and common pathways between UC and colon cancer

**Figure 1:**
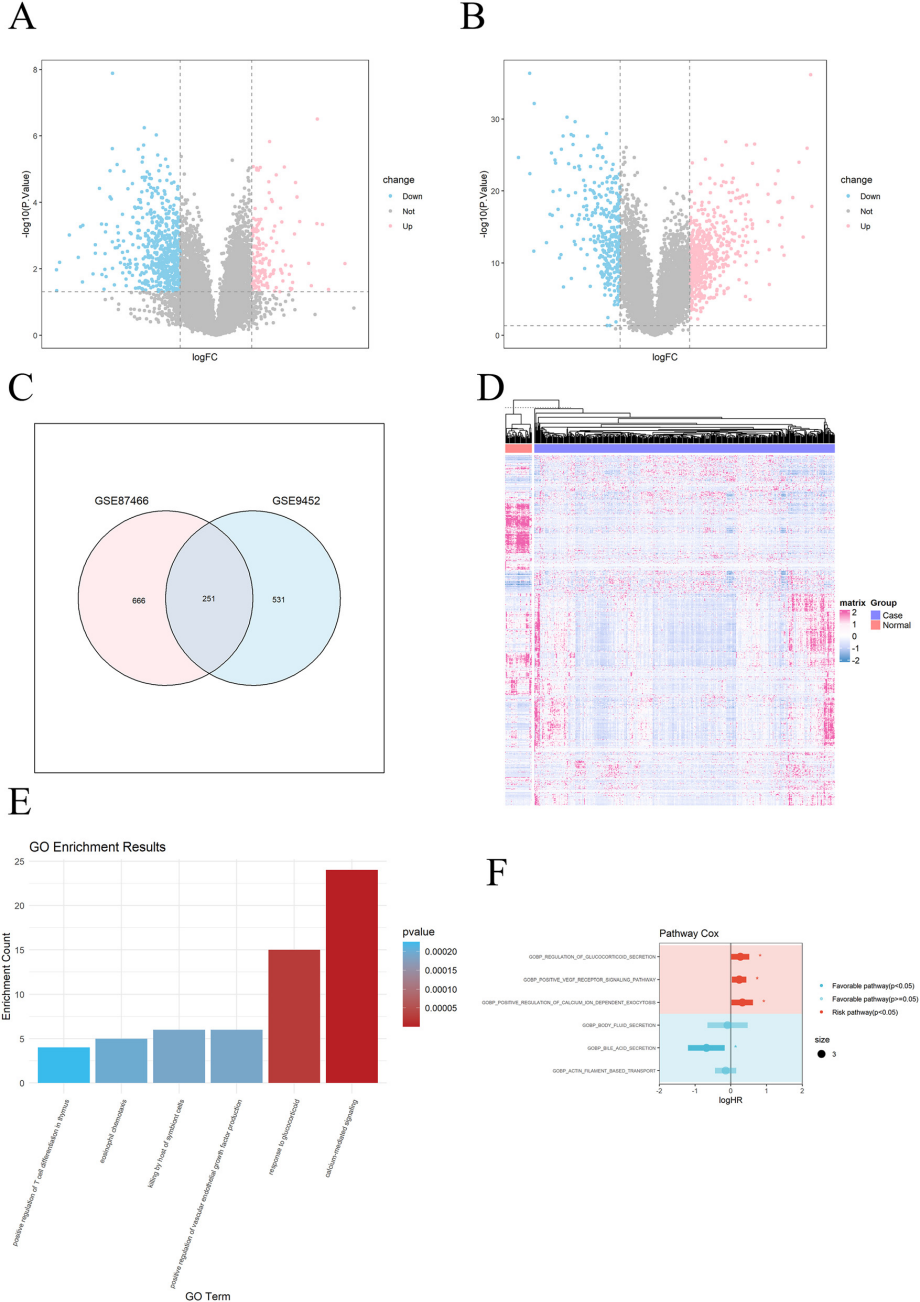
Screening of differentially expressed genes (DEGs). (A) DEGs volcano map of GSE9452 cohort. (B) DEGs volcano map of GSE87466 cohort. (C) Venn plot of merged DEGs in the GSE87466 and GSE9452 cohorts. (D) Expression of merged DEGs in colon cancer. (E) Pathway enrichment analysis of UC. (F) univariate cox analysis of pathways in colon cancer.

917 differentially expressed genes (DEGs) were identified in the GSE87466 cohort, including 596 upregulated genes and 321 downregulated genes (Figure 1A). 782 differentially expressed genes (DEGs) were identified in the GSE9452 cohort, including 554 upregulated genes and 228 downregulated genes (Figure 1B). Merge DEGs and remove duplicate values, resulting in a total of 1,160 differentially expressed genes (Figure 1C). The heat map indicates that these DEGs also differ in the colon cancer cohort (Figure 1D).

Screening three common pathways from the pathway analysis results of UC and colon cancer. Pathway analysis of UC (UC) shows calcium mediated signaling, response to glucocorticoid, positive regulation of vascular endothelial growth factor production have high significance and enrichment rate (Figure 1E). Pathway analysis of colon cancer indicates that positive-regulation of calcium-ion-dependent exocytosis, gobp-regulation of glycoporticoid secretary and positive-regulation of endothelial cell chemotaxis by vegf-activated vascular endothelial growth factor-receptor signaling pathway are risk pathway associated with death (Figure 1F). This study used vascular endothelial growth factor for subsequent research.

### Clinical characteristics and immune analysis of three expression groups

**Figure 2:**
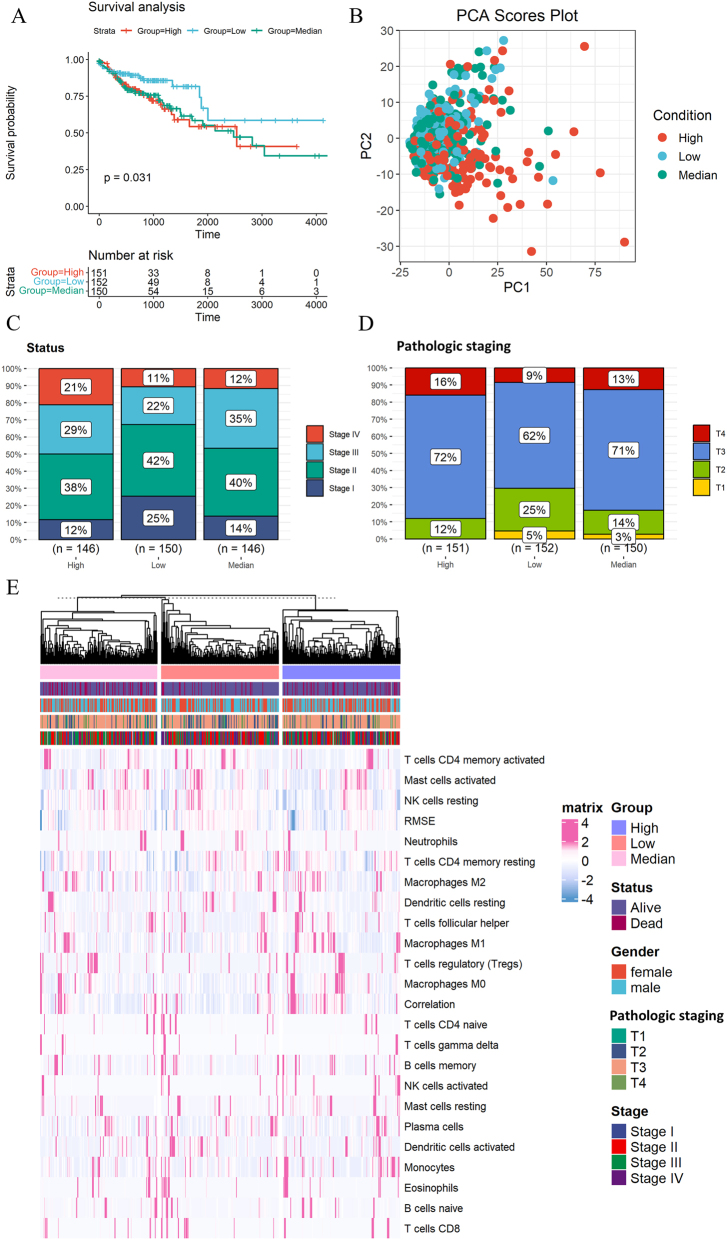
Clinical characteristics of the three expression groups. (A) Survival analysis of high, medium, and low groups. (B) PCA of high, medium, and low groups. (C) Clinical stage in high, medium, and low groups. (D) Pathologic staging in high, medium, and low groups. (E) Expression of immune cells in high, medium, and low groups.

The GSVA scores of all colon cancer patients were divided into three equal groups. The high expression group consists of patients with GSVA scores in the highest third, the medium expression group consists of patients with GSVA scores in the middle third, and the low expression group consists of patients with GSVA scores in the lowest third. Survival analysis showed that the survival rate of the high and median group was significantly lower than that of the low expression group (Figure 2A). PCA indicates that there are differences in gene expression among the high expression group, the medium and low expression group (Figure 2B). The mortality rate of patients in the medium expression group was higher than that in the other two groups (Supplement Figure 2A), and there was no significant gender bias in the three groups (Supplement Figure 2B). The proportion of stage III and stage IV in the high expression group was higher than that in the other two groups (Figure 2C). The pathologic staging of T3 and T4 in the high and median expression group was higher than that in the low expression group (Figure 2D). The content of some immune cells in the high expression group is higher than that in the other two groups (Figure 2E).

**Figure 3:**
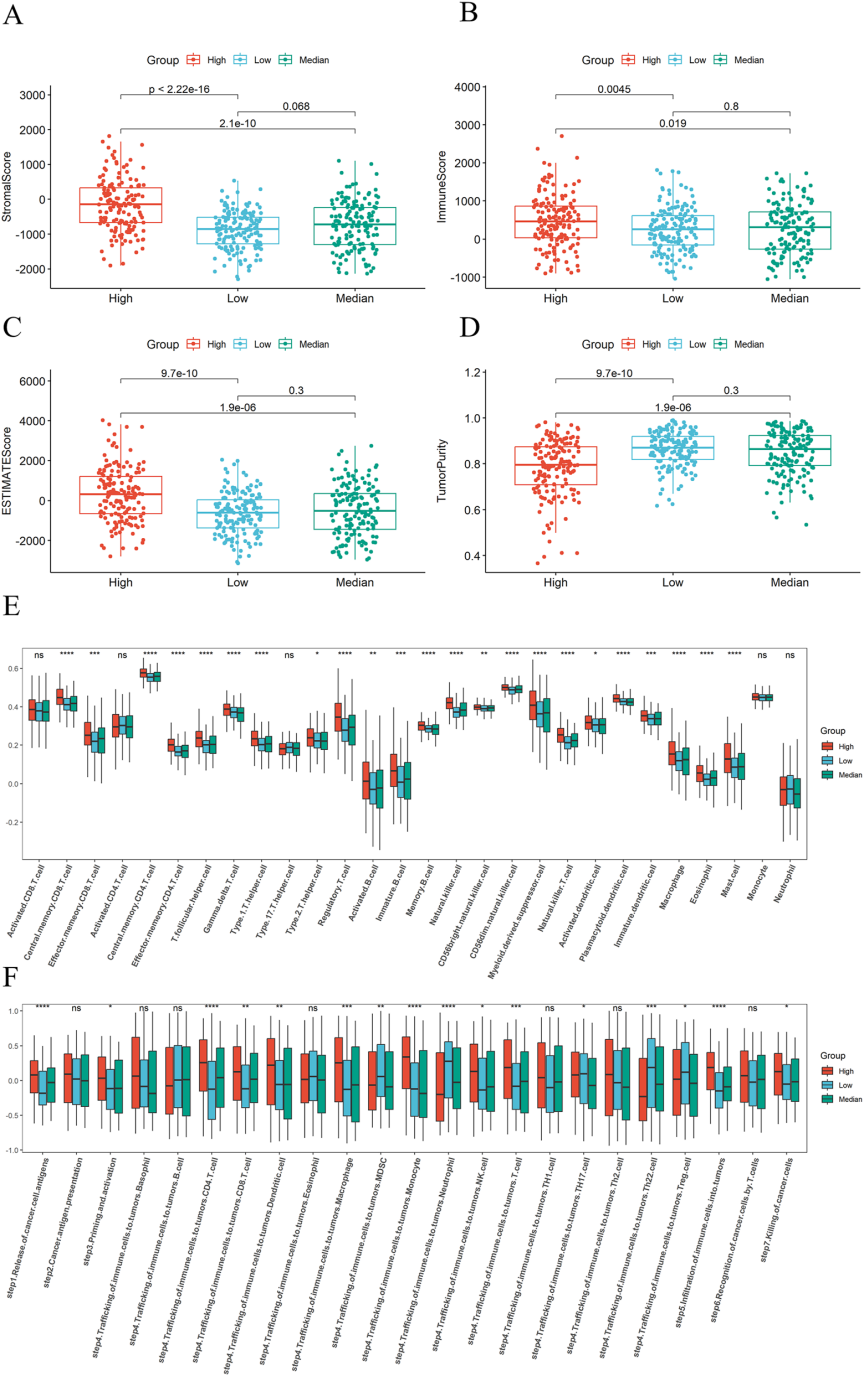
Immune analysis of the three expression groups. (A) Differences in stromal scores among high, medium, and low expression groups. (B) Differences in immune scores among high, medium, and low expression groups. (C) Differences in ESTIMATE scores among high, medium, and low expression groups. (D) Differences in tumor purity among high, medium, and low expression groups. (E) Differences in lymphocytes content among high, medium, and low expression groups. (F) Differences in steps of cancer immune cycle among high, medium, and low expression groups.

Tumor stromal plays an important role in the growth, diffusion, and metastasis of tumors [19]. Immune score refers to an indicator obtained through quantitative analysis, which reflects the richness and activity of immune cells in the tumor microenvironment. The purity of tumors is of great significance for the research and treatment of cancer, as it directly affects the understanding of tumor biological characteristics and the formulation of subsequent treatment strategies. Tumor microenvironment analysis showed that the stromal score of the high expression group was higher than that of the medium and low expression group (Figure 3A), the immune score of the high expression group was higher than that of the medium and low expression group (Figure 3A), and the tumor purity of the medium and low expression group was higher than that of the high expression group (Figure 3C and D). The content of lymphocytes is highest in the high expression group, followed by the medium expression group, and then the low expression group (Figure 3E). Most of the steps in the cancer immune cycle are more active in the high expression group, but there are also individual steps such as Traffic of immune cells to tumors Neutrophil, Traffic of immune cells to tumors Th22 Cells are more active in the low expression group (Figure 3F).

### Construction and validation of risk model

**Figure 4:**
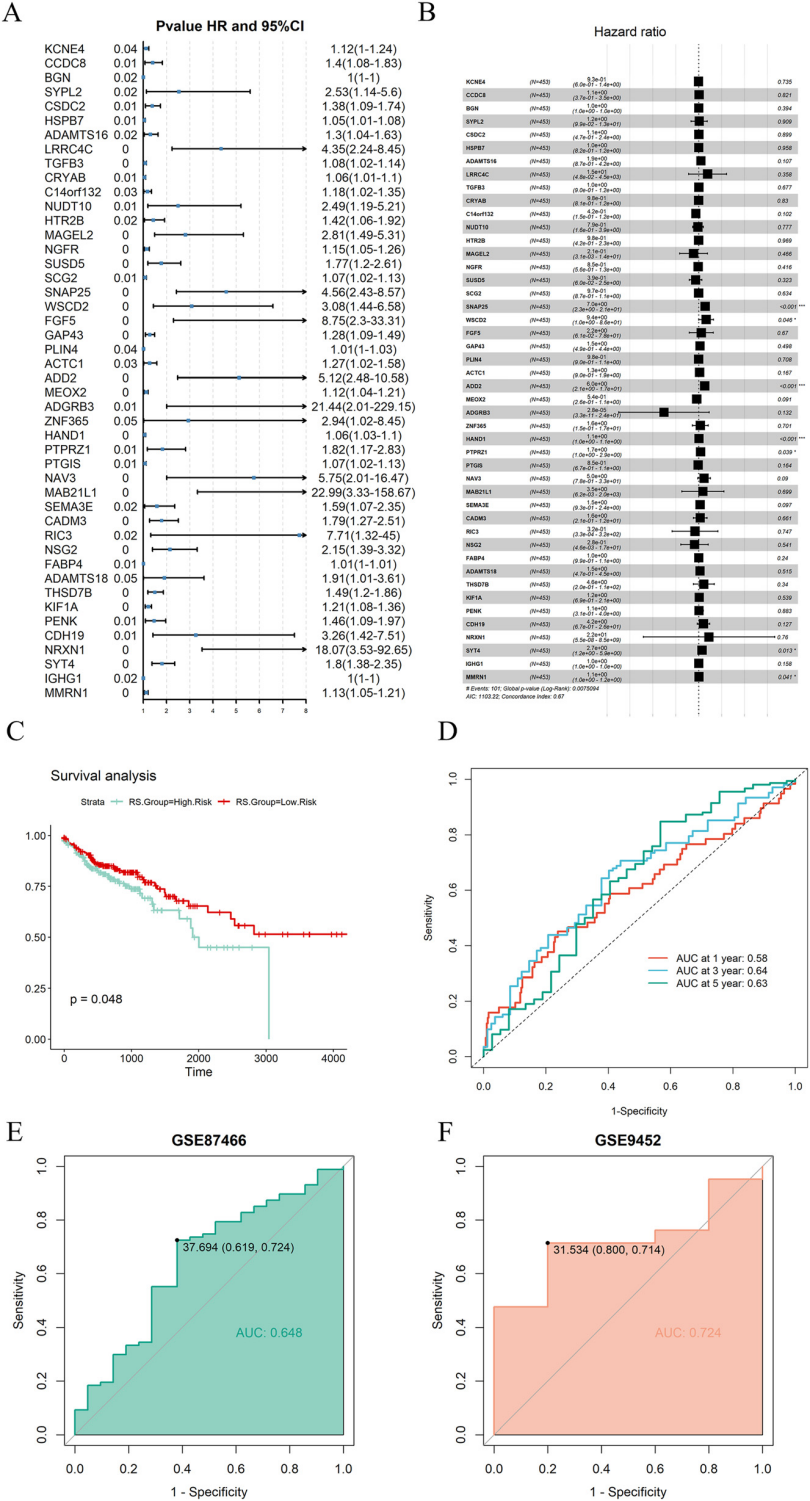
Construction and validation of the risk model. (A) Forest plot of univariate cox results. (B) Forest plot of multivariate cox results. (C) Survival analysis of high risk and low risk groups. (D) ROC curve of risk model for one year, three years, and five years. (E) Validation of risk model in UC cohort GSE87466. (F) Validation of risk model in UC cohort GSE9452.

From the immune microenvironment and immune cell composition, it can be seen that there is no significant difference between the medium and low expression groups, while there are significant differences between the high expression group and the medium/low expression groups. Therefore, when selecting risk genes, we combined the medium and low expression groups into one group and only analyzed the differential genes between the high expression group and the combined group. We analyzed a total of 1916 DEGs between high, medium, and high, low group, univariate cox yielded 46 risk genes (Figure 4A), while multivariate cox yielded 7 gene signatures (Figure 4B). A risk model was established based on 7 gene signatures, and survival analysis showed that the survival rate of the high risk group was significantly lower than that of the low risk group (Figure 4C). The ROC curve showed that the model had the best predictive effect on 3-year survival rate, followed by 5-year and finally 1-year (Figure 4D). We validated this model in the cohorts of UC patients (GSE87466, GSE9452), and the ROC curve showed that the model also had good predictive ability for the occurrence of disease (Figure 4E and F).

### Analysis of clinical characteristics of risk models and construction of nomogram

**Figure 5:**
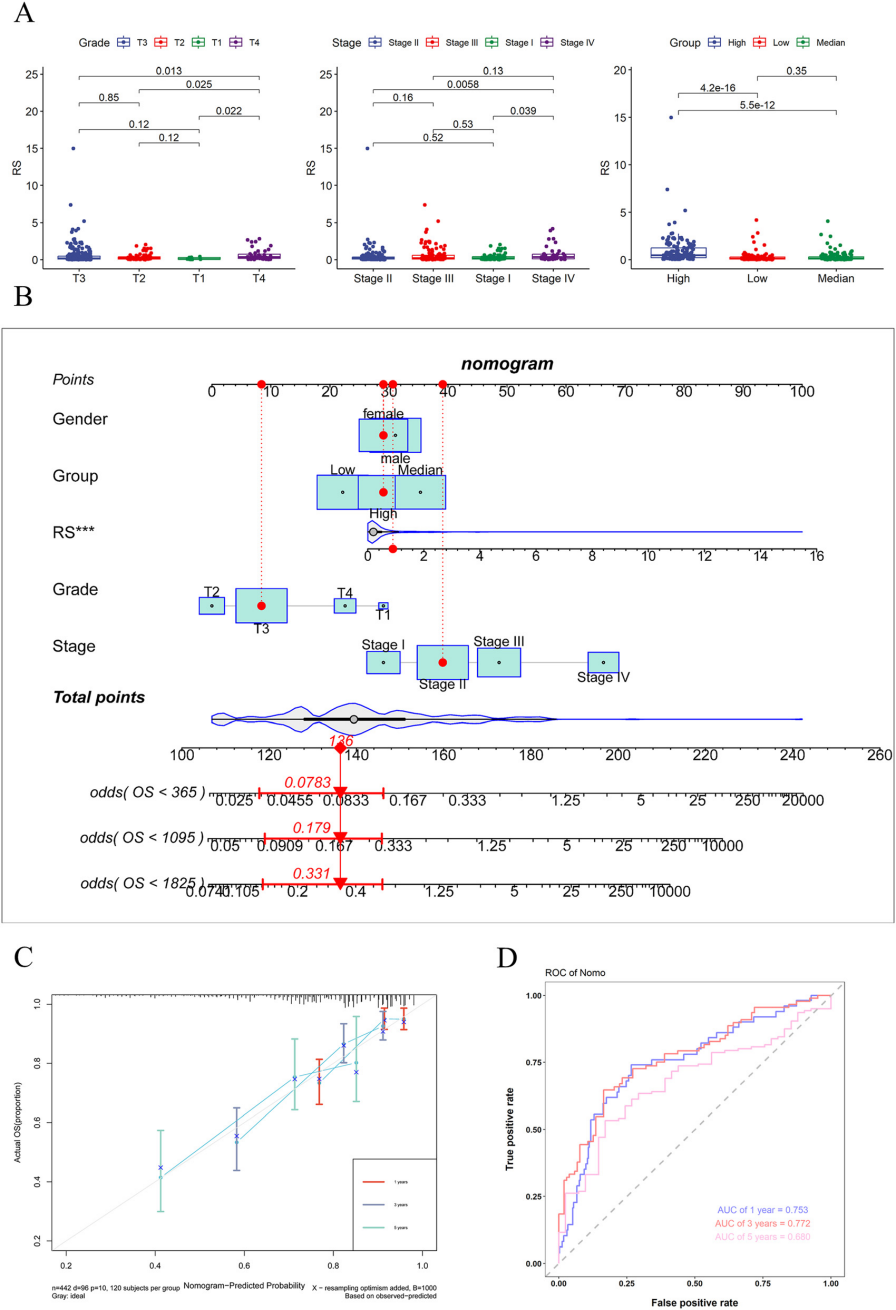
Nomogram of the risk model. (A) Differences in risk scores of clinical features and differences in risk scores among high, medium, and low expression groups. (B) Nomogram of clinical features and risk scores. (C) One year, three year, and five year calibration curves for nomogram. (D) One year, three year, and five year ROC curve of nomogram.

The risk scores of pathologic staging T3 and T4 are higher than those of T1, T2, the risk scores of clinical stage III, and Stage IV, with higher risk scores than Stage I and Stage II. The risk score of the high expression group is significantly higher than that of the medium and low expression group (Figure 5A). The three genes with the highest mutation rates in the high risk and low risk groups are TP53, APC, and TTN. However, the mutation rates in the high risk group are higher than those in the low risk group (Supplement Figure 3A and B). We collected information on the wild-type and mutation types of these three genes and found that the risk score of TTN mutation type is significantly higher than that of wild-type, indicating that individuals carrying TTN mutations have a higher risk of developing colon cancer (Supplement Figure 3C). The tumor stromal score and immune score of the high risk group are higher, but the tumor purity is lower (Supplement Figure 4). We included clinical features and risk scores in the nomogram and found that risk scores were significant prognostic factors (Figure 5B). The calibration curves of the nomogram for one year, three years, and five years are relatively close to a straight line at a 45 degree angle and the area under the ROC curve is 0.753, 0.772, and 0.680, indicating that the model has good accuracy (Figure 5C and D).

### Characteristics of gene signatures

**Figure 6:**
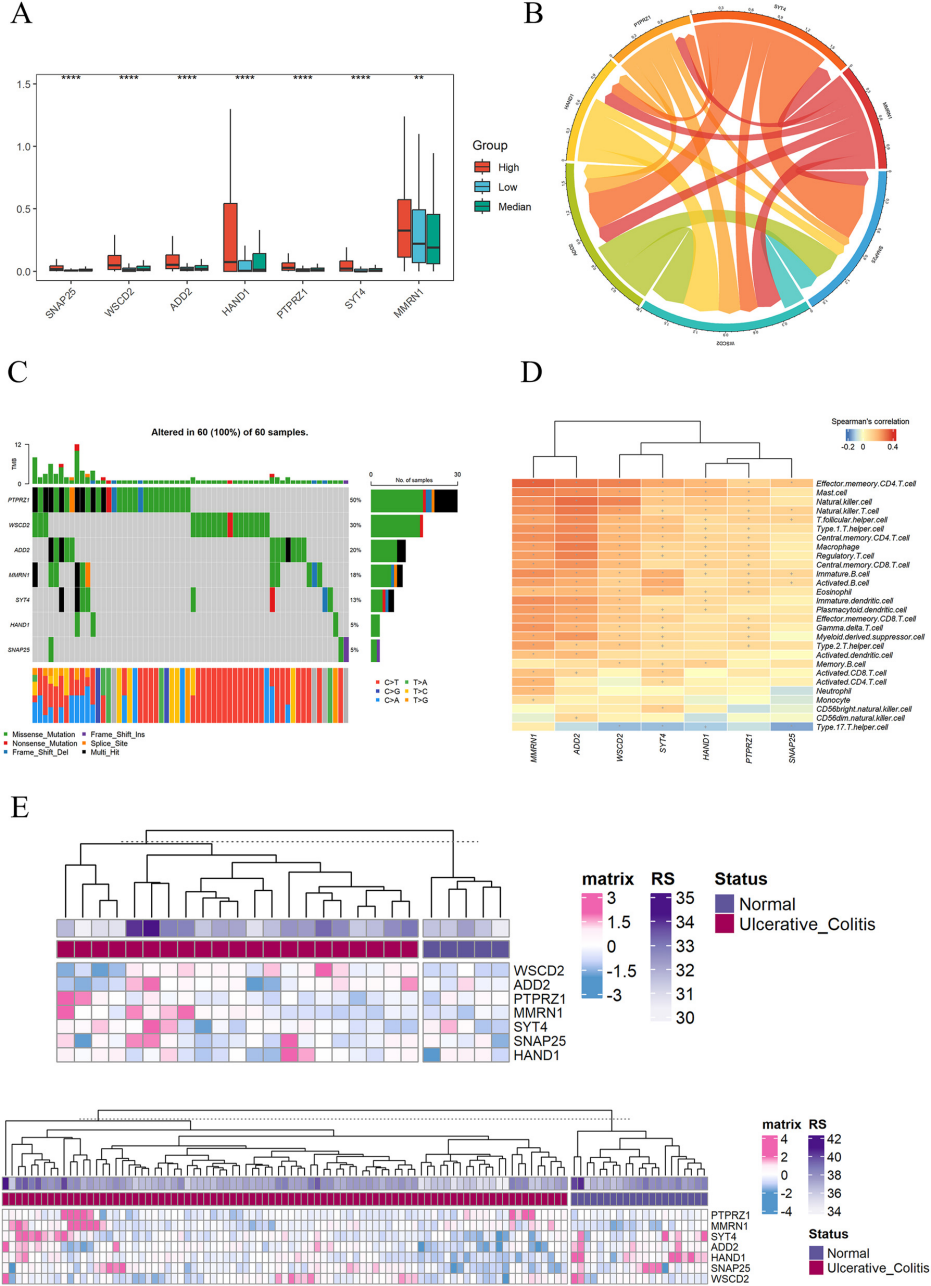
Expression patterns of the gene signatures. (A) Expression differences in gene signatures among high, medium, and low expression groups. (B) Interactions between gene signatures. (C) Mutation of gene signatures in colon cancer samples. (D) The correlation between gene signatures and immune cells (+represents significance less than 0.05, * represents significance less than 0.01). (E) Expression of gene signatures in UC cohorts.

The expression of gene signatures in the high expression group is higher than that in the medium expression group and higher than that in the low expression group (Figure 6A). The interaction analysis among gene signatures reveals that the interaction between SYT4 and SNAP25 is the strongest, followed by SYT4 and ADD2 (Figure 6B), Mutation data analysis shows that the highest proportion of PTPRZ1 gene mutations is 50 %, followed by WSCD2 and ADD2 at 30% and 20 %, respectively (Figure 6C). Most immune cells are positively correlated with gene signatures, but the activation and proliferation of Type 17 T helper (Th17) cell are negatively correlated with the expression of gene signatures (Figure 6D). Although gene signatures were screened based on colon cancer expression data, the regulation of vascular endothelial growth factor related pathways is also more active in UC, therefore, we analyzed the expression of gene signatures in UC, and found that PTPRZ1 and MMRN1 genes have higher expression, while HAND1 gene expression is lower (Figure 6E).

### Predicting the effectiveness of immunotherapy

**Figure 7:**
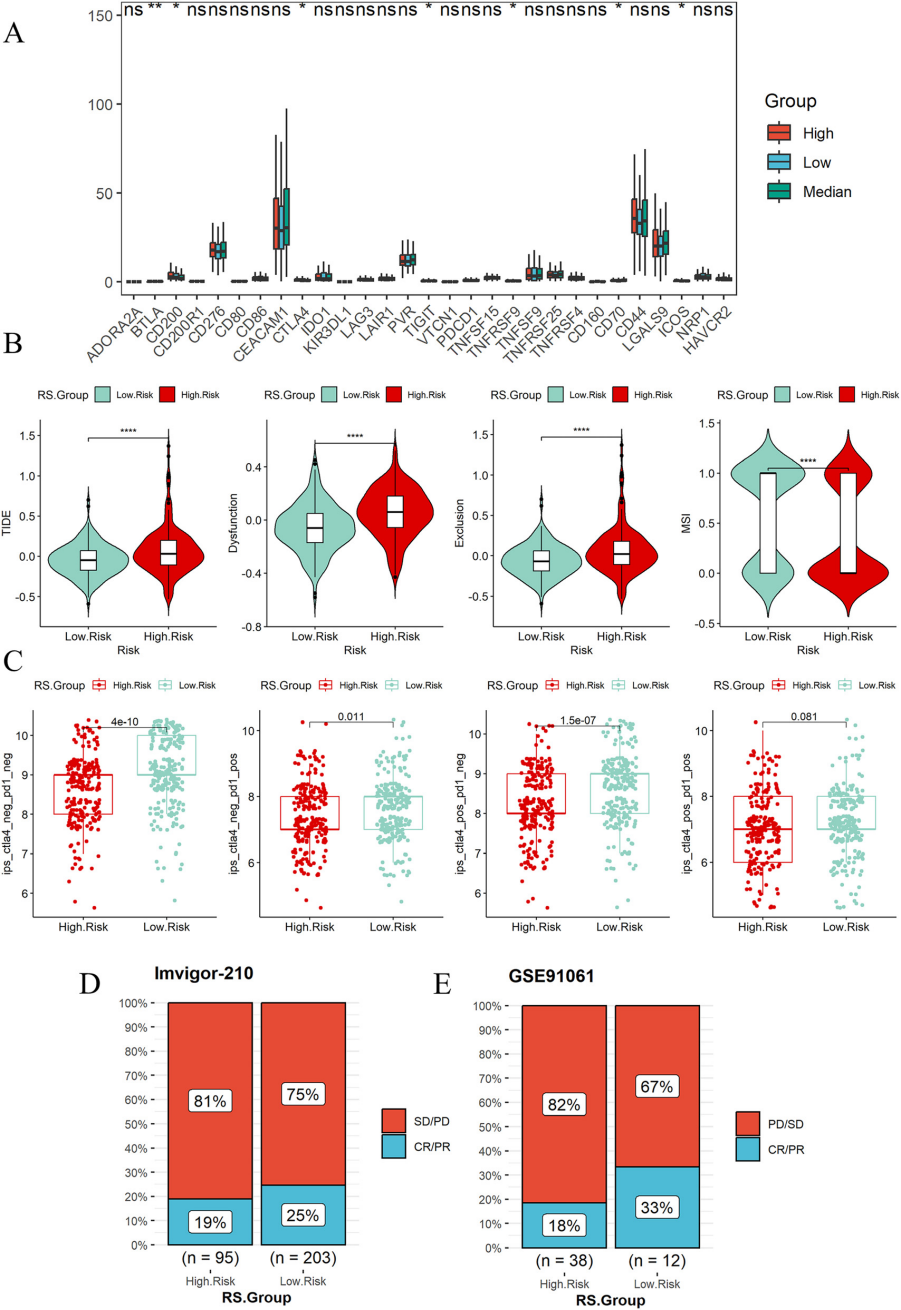
Immune analysis. (A) Expression differences of immune checkpoints in high, medium, and low expression groups. (B) Differences in immune dysfunction, immune exclusion, and microsatellite instability between high risk and low risk groups. (C) Differences in IPS scores between high risk and low risk groups. (D) Validation of immune prediction for risk models.

The immune checkpoint expression in the high expression group is more active than that in the medium and low expression group (Figure 7A). Except for CD44, most immune checkpoints have no difference in expression between the high and low-risk groups (Supplement Figure 5A). The immune dysfunction and immune exclusion scores of the high risk group are higher than those of the low risk group, microsatellite instability expression signature (MSI) of the low risk group was higher than that of the high risk group (Figure 7B). Immune dysfunction score of the high expression group is higher than that of the medium expression group, immune dysfunction score of the medium expression group was higher than that of the low expression group, and the immune exclusion score of the high expression group is higher than that of the medium and low expression group. Microsatellite instability expression signature (MSI) in the high expression group is significantly lower than that in the medium and low expression group (Supplement Figure 5B). We used immunophenotypic scores (IPS) to predict the response to immunotherapy, and the results showed that the immunotherapy effect in the low risk group was better than that in the high risk group (Figure 7C), and the immunotherapy effect in the medium and low expression group was better than that in the high expression group (Supplement Figure 5C). Risk model was constructed in the Imvigor-210 and GSE91061 immunotherapy cohorts, which verified that the immunotherapy effect of the low risk group was better (Figure 7D).

### Drug sensitivity analysis

**Figure 8:**
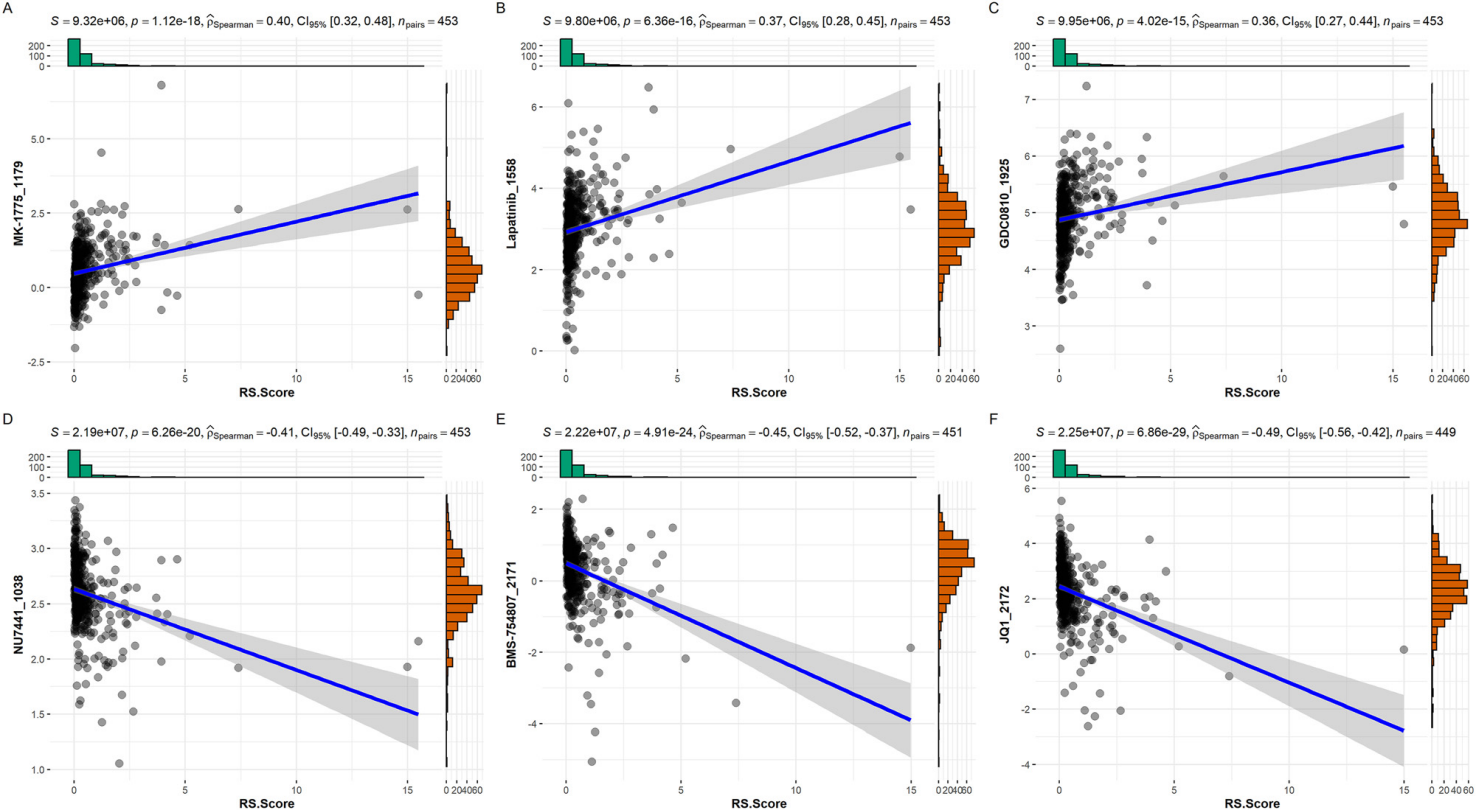
Correlation analysis between the risk model and drug sensitivity. (A) Correlation analysis between IC50 of MK-1775_1179 and risk score. (B) Correlation analysis between IC50 of Lapatinib_1558 and risk score. (C) Correlation analysis between IC50 of GDC0810_1925 and risk score. (D) Correlation analysis between IC50 of NU7441_1038 and risk score. (E) Correlation analysis between IC50 of BMS-754807_2171 and risk score. (F) Correlation analysis between IC50 of JQ1_2172 and risk score.

The IC50 of MK-1775_1179, Lapatinib_1558, GDC0810_1925 is positively correlated with risk scores (Figure 8A–C). NU7441_1038, BMS-754807_2171, JQ1-2172 have a negative correlation between IC50 and risk scores (Figure 8D–F).

## Discussion

In the early stages of colon cancer, symptoms may be completely absent, making regular screening essential for early detection and timely treatment. Patients with long-term ulcerative colitis (UC) face an increased risk of developing colon cancer, primarily due to chronic inflammation-induced damage to colon cells and mutations arising during the repair process [20]. Therefore, individuals with UC should undergo routine intestinal surveillance and cancer screening to identify and address potential malignancies as early as possible [21]. Identifying common signaling pathways involved can reveal specific biomarkers or pathways in UC patients that contribute to colon cancer development, aiding in the creation of early diagnostic tools and preventive strategies. Early detection greatly enhances colon cancer survival rates, underscoring the importance of this study.

VEGF plays a critical role in the pathological processes of both ulcerative colitis (UC) and colon cancer. In chronic inflammatory bowel diseases like UC, VEGF can worsen inflammation by promoting angiogenesis, thereby supporting the growth and persistence of inflamed tissue [22]. Elevated VEGF levels are associated with the inflammatory activity of ulcerative colitis (UC) and may serve as a marker of disease severity. In colon cancer, VEGF promotes tumor angiogenesis, supplying nutrients and oxygen that support tumor growth and metastasis, thereby facilitating tumor progression. The expression level of VEGF is closely linked to tumor invasiveness, staging, prognosis, and response to treatment [23], 24]. This is also the reason why the high expression group of VEGF has a lower survival rate, higher clinical stage, and poorer prognosis.

To assess the risk of colon cancer and differentiate subtypes with varying prognoses and treatment responses, we developed a risk model based on gene signatures identified from differentially expressed genes between high and medium expression groups, as well as between high and low expression groups [25]. The high risk group had lower survival rate, higher clinical stage and pathologic staging, higher gene mutation rate, higher immune stromal and immune score, indicating a higher degree of immune infiltration in the high risk group, which also reflects the complexity of the tumor microenvironment in this subtype, including the interaction between immune cells and matrix cells [26]. This complex microenvironment may promote tumor development and immune escape [27], which is one of the reasons for poor prognosis in the high risk group. We found that this risk model can not only predict the prognosis of colon cancer but also accurately estimate the incidence of UC. It aids in the early identification of high-risk patients, enabling timely intervention to reduce the risk of disease progression and colon cancer. Additionally, the model reveals shared pathogenic mechanisms and risk factors between the two diseases, offering new insights for basic research and facilitating the development of novel treatments and drugs.

SYT4, a protein located on the synaptic vesicle membrane, is involved in regulating the release of neurotransmitters. When the nerve cell membrane is stimulated, SYT4 binds with calcium ions, promoting the release of neurotransmitters [28]. SNAP25, another gene involved in regulating neurotransmitter release, combines with other t-SNARE proteins to form a SNARE complex, facilitating the release of neurotransmitters [29]. The strong interaction between these two proteins suggests they may play a role in regulating intercellular communication within the tumor microenvironment. Elevated expression levels of SYT4 and SNAP25 could enhance signal transduction between cancer cells, thereby accelerating cell migration and invasion. This strong interaction may also facilitate signaling between tumor cells and immune cells, thereby upregulating the levels of chemokines such as CXCL12 and CCL2, helping tumors evade immune surveillance and promoting immune escape. This signaling pathway has been validated in prostate cancer [30], but its specific mechanism in colon cancer has not yet been fully explored. Future research will require more experimental studies to validate its precise mechanisms.

The mutation rate of PTPRZ1 is the highest among genetic sigantures. PTPRZ1 is a protein tyrosine phosphatase that typically involves the regulation of cell proliferation, differentiation, and migration [31]. Its high mutation rate may affect these biological processes, promoting the proliferation, invasion, and metastasis of cancer cells [32]. This high mutation rate may also reflect the underlying heterogeneity of colon cancer. SNAP25 (Synaptosomal-associated protein 25) is primarily expressed in neuronal cells and is involved in the regulation of neurotransmitter release. Th17 cells represent a key subset of helper T cells that play a pivotal role in modulating inflammatory responses and contributing to the progression of certain autoimmune diseases [33]. SNAP25 is negatively correlated with Type 17 T helper cells, suggesting that SNAP25 may reduce Th17 cell activity by releasing certain neurotransmitters, thereby inhibiting the differentiation or function of Th17 cells, which could promote the spread and invasion of tumor cells [34]. Alternatively, the inflammatory factors produced by Th17 cells might suppress neurotransmitter release, thereby reducing SNAP25 expression and inhibiting tumor growth [35].

In colon cancer, high microsatellite instability (MSI-H) is an important molecular feature that reveals specific biological characteristics and clinical behavior of the tumor [36]. MSI is more prevalent in the low-risk group, suggesting a lower rate of lymph node involvement, reduced distant metastasis, and a more favorable prognosis. Additionally, the low-risk group exhibits a better response to immune checkpoint inhibitors. This is likely because MSI-high (MSI-H) tumors typically carry a higher mutation burden, leading to the generation of more neoantigens, which enhances the immune system’s ability to recognize and eliminate cancer cells [37]. The high risk group had higher scores for immune exclusion and immune dysfunction, indicating that their immune system was suppressed or malfunctioned in resisting tumors, tumor cells prevented immune cells from entering the tumor microenvironment, thereby evading immune attacks. IPS also proved that the immune therapy effect of the high risk group was worse.

The IC50 of MK-1775_1179 is positively correlated with the risk score to the greatest extent, indicating that it has a better therapeutic effect on the low risk group. MK-1775 (chemically known as Adaposertib) is an experimental anti-cancer drug that mainly works by inhibiting the Wee1 kinase [38]. In cancer cells, the expression and activity of Wee1 are often elevated, enabling the cells to bypass DNA damage checkpoints and continue dividing, which contributes to tumor progression. MK-1775, a selective Wee1 kinase inhibitor, disrupts this protective mechanism by forcing cancer cells to proceed through the cell cycle without repairing DNA damage, ultimately resulting in cell death. However, MK-1775 remains under clinical investigation and has not yet received official approval for therapeutic use in any country. The IC50 of JQ1 is negatively correlated with the risk score to the greatest extent, indicating its better therapeutic effect on the high risk group. JQ1 is an experimental small molecule inhibitor that inhibits the function of BET protein by binding to the bromine domain of BET protein, blocking its binding to acetylated histones [39]. This inhibitory effect can reduce the expression of certain oncogenes, including genes that play critical roles in various cancers such as hematological cancer and solid tumors.

The innovation of this study lies in the development of a universal risk model applicable to both ulcerative colitis (UC) and colon cancer (CC), based on their shared molecular pathways. From an immunological perspective, the model helps explain why colon cancer patients with higher risk scores tend to have worse prognoses. It also enables the identification of individuals at high risk of developing UC and those likely to have poor outcomes in colon cancer, thereby supporting early clinical intervention and prevention strategies for UC, as well as the implementation of personalized screening and monitoring plans for high-risk colon cancer patients. Additionally, by combining the UC risk prediction and colon cancer prognosis assessment into a single model, this approach simplifies the analysis process and enhances efficiency compared to the conventional use of separate models.

This study also has certain limitations. Merging the medium and low expression groups into one group for risk gene screening helps improve the model’s simplicity, reduce statistical noise, and better study the characteristics of the high expression group. However, since the characteristics between the medium and low expression groups are not completely overlapping, in practical application, this approach may mask potential clinical heterogeneity and fail to provide precise clinical decision-making guidance for the medium and low expression group. When validating with the GSE87466 and GSE9452 datasets, there may be biases in the patient populations, such as differences in age, gender, and disease severity. Therefore, these datasets may not fully represent all ulcerative colitis patients. The cancer datasets used for immunotherapy response prediction, GSE91061 and the Imvigor-210 cohort, exhibit certain biological heterogeneity, including differences in tissue sampling methods, genetic backgrounds of patient populations, and inconsistencies in clinical annotation standards. This heterogeneity may reduce the generalizability of the model and complicate clinical interpretation; thus, greater caution should be exercised when applying the model in clinical practice. Although the risk model we have constructed demonstrates good predictive ability, our validation has been based on existing public databases and lacks independent clinical experiments. The gene markers we identified need to be further validated for their expression in colon cancer patients through methods such as qPCR, gene knockout, and overexpression experiments. The role of the interaction between SYT4 and SNAP25 in promoting tumor immune evasion still requires experimental validation. In the future, the correlation between the IC50 of drugs in the drug sensitivity analysis and the risk score can be further validated *in vitro* or *in vivo* to better understand the therapeutic effects of these drugs on colon cancer patients.

## Conclusions

We obtained three subtypes of colon cancer related to vascular endothelial growth factor, a risk model applicable to UC and colon cancer, as well as gene signatures of colon cancer. We conducted clinical features and immune analysis on the three expression subtypes and two risk subtypes. Our research results provide new ideas for the diagnosis and treatment of UC and colon cancer. Although our findings offer potential new insights for the diagnosis and treatment of ulcerative colitis and colon cancer, further experimental and clinical validation is needed to confirm their clinical applicability in the future.

## Supplementary Material

Supplementary Material

Supplementary Material

Supplementary Material

Supplementary Material

Supplementary Material

Supplementary Material
