# Supplementary figures and images for: Prognostic value of vascular endothelial growth factor subtypes and risk models constructed based on the common pathway of ulcerative colitis and colon cancer
Source: Open Med (Wars). 2026 Feb 12;21(1):20251245. doi: 10.1515/med-2025-1245 (PMC12917561; doi:10.1515/med-2025-1245)

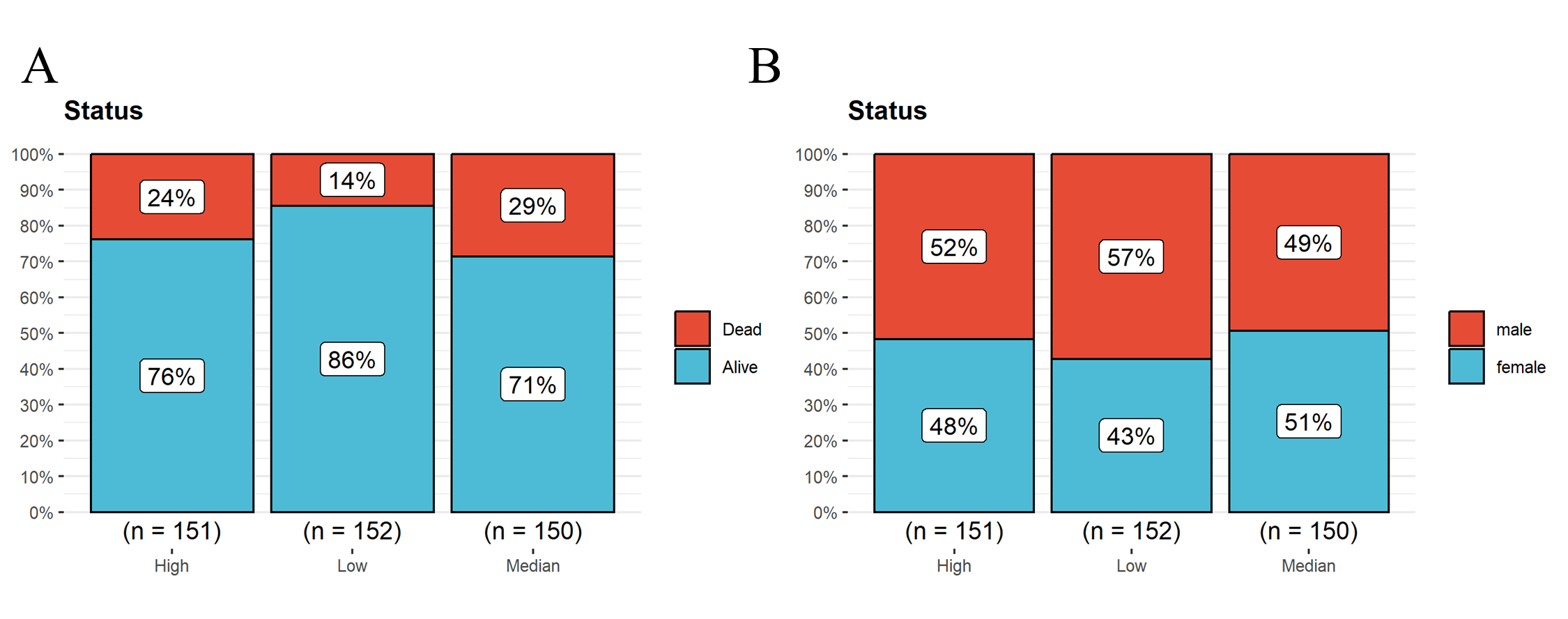

Supplement: Supplementary file 2 — Supplementary Material [file j_med-2025-1245_suppl_002.zip › j_med-2025-1245_suppl_002.tif]

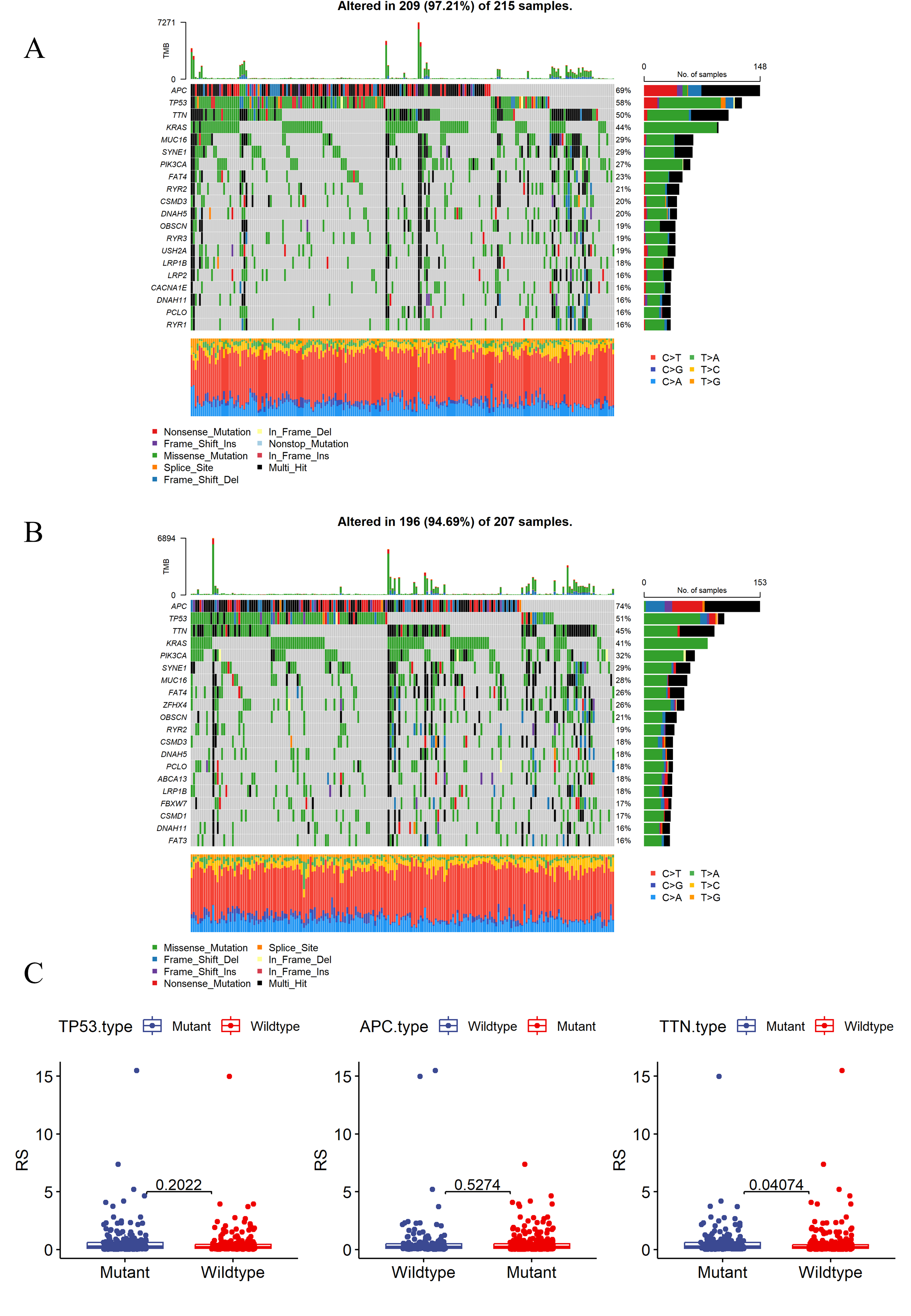

Supplement: Supplementary file 3 — Supplementary Material [file j_med-2025-1245_suppl_003.zip › j_med-2025-1245_suppl_003.tif]

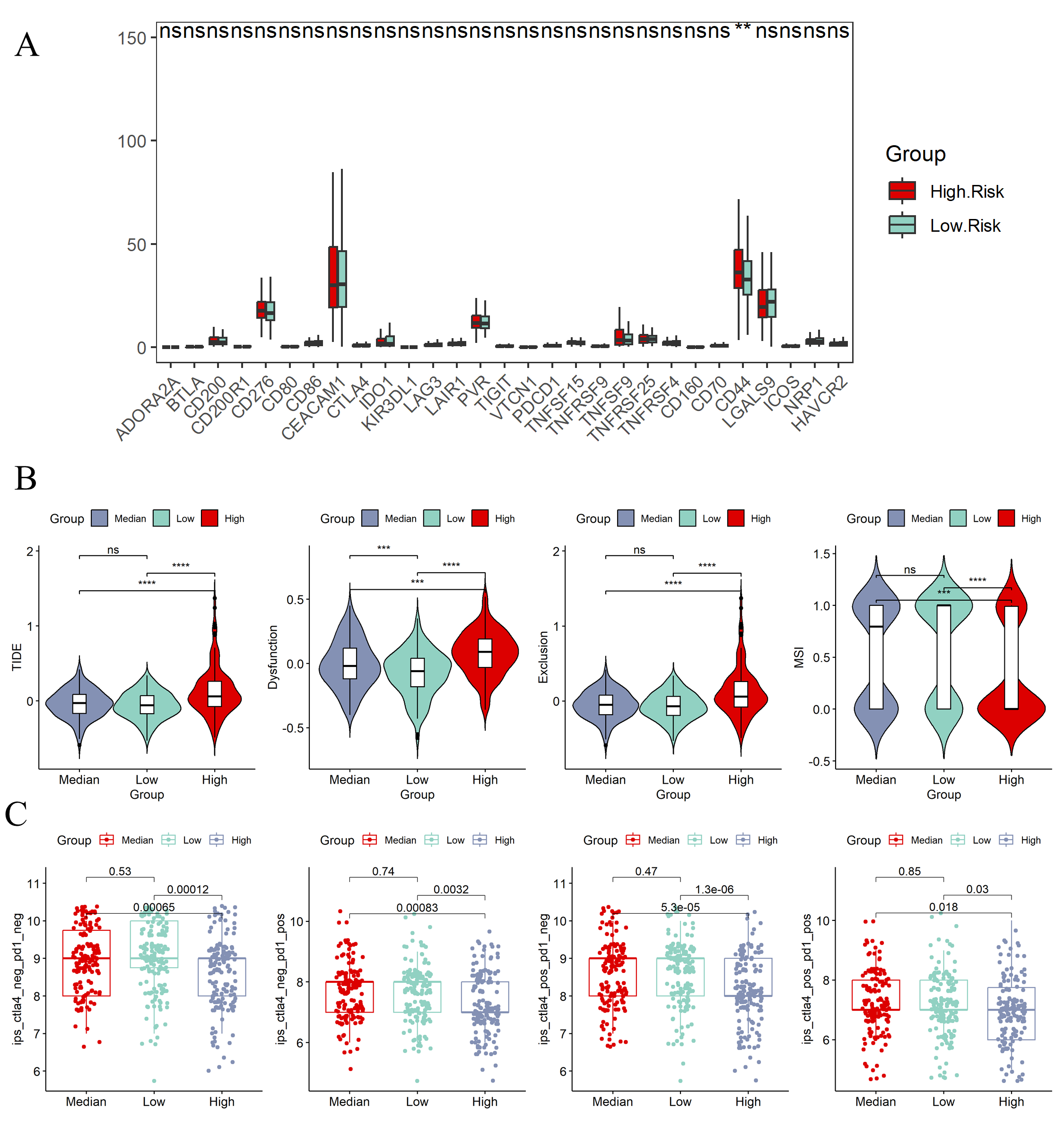

Supplement: Supplementary file 5 — Supplementary Material [file j_med-2025-1245_suppl_005.zip › j_med-2025-1245_suppl_005.tif]
