# Supplementary material for: Prognostic value of vascular endothelial growth factor subtypes and risk models constructed based on the common pathway of ulcerative colitis and colon cancer
Source: Open Med (Wars). 2026 Feb 12;21(1):20251245. doi: 10.1515/med-2025-1245 (PMC12917561; doi:10.1515/med-2025-1245)
Supplement: Supplementary file 6 — Supplementary Material [file j_med-2025-1245_suppl_006.docx]

Supplement Figure. 1 Research workflow diagram

Supplement Figure. 2 (A) Survival status of patients in high, medium, and low groups. (B) Gender distribution of patients in high, medium, and low groups.

Supplement Figure. 3 (A) Somatic mutations in high risk groups. (B) Somatic mutations in low risk groups. (C) Differences in risk scores between wild type and mutant types of TP53, APC, and TTN

Supplement Figure. 4 Analysis of differences in immune microenvironment between high risk and low risk groups

Supplement Figure. 5 (A) Differences in immune checkpoint expression among high, medium, and low expression groups. (B) Differences in immune dysfunction, immune exclusion, and microsatellite instability among high, medium, and low expression groups. (C) Differences in IPS scores among high, medium, and low expression groups
